# African perspectives: modern complexities of emerging, re-emerging, and endemic zoonoses

**DOI:** 10.7189/johg.08.020310

**Published:** 2018-12

**Authors:** Abdulazeez Muzemil, Olubunmi Gabriel Fasanmi, Folorunso Oludayo Fasina

**Affiliations:** 1 Faculty of Veterinary Medicine, Usmanu DanFodio University, Sokoto, Nigeria; 2 Department of Animal Health, Federal College of Animal Health and Production Technology, Ibadan, Nigeria; 3 Department of Veterinary Tropical Diseases, University of Pretoria, South Africa & Food and Agriculture Organization of the United Nations, Dar es Salaam, United Republic of Tanzania

Recent events have shown that public health, animal health and national economies have been threatened, globally, by the increased occurrence of emerging and re-emerging infectious diseases (ErEIDs) [1]. Specifically, land use change *cum* agricultural practices, surging human demographic, pathogen evolution (antimicrobial resistance), failure of public health systems, global travel and more global interconnectedness in spatial and temporal dimensions have driven these threats [2]. Other aggravating factors include: ecological changes, incursion into previously uninhabited areas, changes in human behavior, environmental degradation, international trade, technology and industry, antimicrobial misuse, and deficiencies in public health infrastructure and decision-making [1-3]. Major ErEIDs − including zoonoses − have been reported in the last two decades including: Bovine Spongiform Encephalopathy, Hendra, Nipah, Severe Acute Respiratory Syndrome (SARS), Highly Pathogenic Avian Influenza (HPAI) H5N1, H5N8 and H7N9 subtypes, West Nile fever, Pandemic H1N1 Influenza, Ebola virus disease (EVD) and Middle East respiratory syndrome coronavirus (MERS-CoV). Many of these diseases have been documented in Africa.

In Africa, 47 of the 54 countries (87%) have reported ErEIDs to the WHO since 1997 (**Table 1**). While several initiatives have been implemented globally to accelerate progress toward a safer world [4], it is yet not clear whether African countries are ready and capable of handling the magnitude and threats associated with ErEIDs [4]. Here, we reviewed plausible reasons and drivers for the upsurge of ErEIDs in Africa and proffer some mitigating measures.

**Table 1:** Reported emerging and re-emerging diseases in humans, Africa*

Country & 2015 mid-year normalized demographic projections	Reported disease
Algeria (43 333 000)	Plague (2003); MERS-CoV (2014)
Angola (32 827 000)	Meningococcal disease (2001); Marburg HFS (2005); Poliomyelitis (2005); Cholera (2006); Poliomyelitis (2008, 2010); Yellow fever (2016)
Botswana (2 416 000)	None reported
Burkina Faso (20 903 000)	Meningococcal disease (2001-2004, 2006, 2007, 2012, 2014); Cholera (2001, 2005); Poliomyelitis (2003); Yellow fever (2003-2005, 2008); Dengue fever (2016); Lassa fever (2017)
Burundi (11 939 000)	Cholera (2002, 2004); Meningococcal disease (2002)
Cameroun (25 958 000)	Meningococcal disease (2001); Cholera (2004, 2010); Yellow fever (2009, 2010,2013); Wild poliovirus (2013, 2014)
Cape Verde (567 000)	Dengue fever (2009); Zika virus infection (2015)
Central African Republic (4 921 000)	Meningococcal disease (2000, 2001, 2014); Shigellosis (2003); Monkey pox (2016); Yellow fever (2008, 2009)
Chad (16 285 000)	Meningococcal infection (2001, 2004, 2005, 2009-2013); Cholera (2001, 2004, 2010); Poliomyelitis (2003, 2007); Hepatitis E (2004, 2017); Yellow fever (2013)
Congo (5 687 000)	None posted
Cote d'Ivoire (26 172 000)	Yellow fever (2001, 2005, 2006, 2008, 2010, 2011); Cholera (2001-2003); Wild poliovirus (2011); Meningococcal infection (2012, 2013); Dengue fever (2017)
Democratic Republic of Congo (89 505 000)	Monkey pox (1997); Meningitis (1998); Cholera (1998, 1999, 2002, 2003, 2011, 2012, 2015); Marburg HF (1999-2001); Acute respiratory syndrome (2002), Influenza (2003), Typhoid fever (2004, 2005); Plague (2005, 2006); Meningococcal infection (2007); Poliomyelitis (2007, 2017); Ebola haemorrhagic fever (2007-2009, 2012, 2014, 2017); Yellow fever (2010, 2013, 2014, 2016)
Djibouti (1 000 000)	Avian influenza (2006, 2007)
Egypt (102 941 000)	Avian influenza (2006, 2007, 2008, 2009, 2010, 2011, 2012); Wild poliovirus (2013); MERS-CoV (2014); Dengue fever (2015)
Equatorial Guinea (1 406 000)	Wild poliovirus (2014)
Eritrea (5 432 000)	None posted
Ethiopia (112 759 000)	Meningococcal infection (2000, 2001, 2002); Anthrax (2000); Poliomyelitis (2005, 2006); Acute watery diarrhoea syndrome (2006); Yellow fever (2013); Wild poliovirus (2013)
Gabon (2 151 000)	Viral haemorrhagic fever (2001); Ebola HF (2001, 2002); Acute haemorrhagic fever (2002)
Gambia (2 293 000)	Meningococcal disease (2001)
Ghana (30 734 000)	Poliomyelitis (2003); Yellow fever (2012); Meningococcal disease (2012, 2013)
Guinea (13 751 000)	Yellow fever (2000, 2001, 2003, 2005, 2008-2010); Cholera (2001, 2005); Meningococcal infection (2012, 2013); Ebola (2014)
Guinea Bissau (2 001 000)	Cholera (2005, 2008)
Kenya (53 492 000)	Leptospirosis (2004); Meningococcal disease (2006); Rift Valley fever (2006, 2007); Wild poliovirus (2013); Yellow fever (2016); Cholera (2017)
Lesotho (2 322 000)	Dysentery (2000)
Liberia (5 104 000)	Yellow fever (2000, 2001, 2004, 2008, 2009); cholera (2002, 2003, 2005); bloody diarrhoea (2003); Ebola HF (2014), Lassa fever (2016); Unexplained clusters of death (2017); Meningococcal septicaemia (2017)
Libya (6 662 000)	None posted
Madagascar (27 691 000)	Cholera (2000); Acute respiratory syndrome (2002); Influenza (2002); Rift valley fever (2008); Wild poliovirus (2014); Plague (2015, 2017)
Malawi (20 284 000)	Cholera (2002); Plague (2002)
Mali (20 284 000)	Cholera (2003-2005); Yellow fever (2005); Ebola VD (2014)
Mauritania (4 784 000)	Crimean-Congo haemorrhagic fever (2003); Cholera (2005); Rift valley fever (2012)
Mauritius (1 274 000)	None posted
Morocco (37 071 000)	Meningococcal disease (2000); Human influenza epidemic (2003)
Mozambique (32 309 000)	Cholera (2002-2004)
Namibia (2 697 000)	Plague (1999); Poliomyelitis (2006)
Niger (24 075 000)	Meningococcal disease (2001-2003, 2006, 2009, 2015); Cholera (2001, 2002, 2004, 2005, 2010); Avian influenza (2006); Rift valley fever (2015, 2016); Hepatitis E (2017)
Nigeria (206 153 000)	Yellow fever (2000); Cholera (2001, 2004, 2005, 2010, 2017); Meningococcal disease (2004, 2009, 2015, 2017); Acute fever & rash syndrome (2005); Avian influenza (2006, 2007); Poliomyelitis (2009); Lead poisoning (2010); Lassa fever (2012, 2016, 2017); Ebola VD (2014); Wild poliovirus & vaccine derived polio (2016); Acute hepatitis E (2017), Monkey pox (2017)
Rwanda (13 087 000)	Meningococcal disease (2000, 2002)
Sao Tome and Principe (218 000)	None posted
Senegal (17 200 000)	Yellow fever (2002, 2005, 2010, 2011); Cholera (2004, 2005); Lead intoxication (2008); Ebola VD (2014); Chikungunya (2015)
Seychelles (96 000)	Chikungunya (2006); plague (2017)
Sierra Leone (8 047 000)	Dysentery (2000); Yellow fever (2003, 2009, 2011); Lassa fever (2014); Cholera (2012); Ebola VD (2014)
Somalia (16 105 000)	Cholera (2000), Meningococcal disease (2001. 2002); Poliomyelitis (2005, 2006); Rift valley fever (2007); Wild poliovirus (2013)
South Africa (58 721 000)	Cholera (2000, 2001, 2003, 2004); Severe acute respiratory syndrome (2003); Unknown disease (2008); Infection from arenaviridae (2008); Rift valley fever (2010)
South Sudan (13 610 000)	Meningococcal disease (2013); Wild poliovirus (2013, 2014); Cholera (2014); haemorrhagic fever syndrome (2016)
Sudan (43 541 000)	Meningococcal disease (2000, 2005, 2006, 2007); Yellow fever (2003, 2005, 2012, 2013); Acute haemorrhagic fever syndrome (2004); Ebola haemorrhagic fever (2004); Shigellosis (2004); hepatitis E (2004); Cholera (2006); Rift valley fever (2007, 2008); Poliomyelitis (2009); Wild poliovirus (2013)
Swaziland (1 439 000)	None posted
Togo (8 384 000)	Poliomyelitis (2003); Yellow fever (2006, 2007); Lassa fever (2016, 2017); Meningococcal disease (2017)
Tunisia (11 903 000)	Novel corona virus (2013); MERSCoV (2013)
Uganda (47 188 000)	Ebola HF (2000, 2001, 2007, 2011, 2012); Cholera (2003); Meningococcal disease (2006, 2007); Marburg disease (2007, 2008, 2012, 2014); Yellow fever (2011, 2016); Typhoid fever (2015)
United Republic of Tanzania (62 775 000)	Cholera (2001, 2015, 2016); meningococcal disease (2002); Rift valley fever (2007)
Zambia (18 679 000)	Plague (2001); Cholera (2003, 2004); Unknown disease (2008); New virus from arenaviridae (2008)
Zimbabwe (17 680 000)	Meningitis (1997); Cholera (1998, 2008, 2009); Viral haemorrhagic fever (1999)

*Only 52 countries were listed on the WHO website. Benin (12 123 000); Republic of Congo (4 706 000); Réunion(France) (897 000); Comoros (870 000); Western Sahara (597 000); Mayotte (France) (273 000); Saint Helena, Ascension and Tristan da Cunha (UK) (4000) were not listed on the WHO website and have no report. Source: Emergencies preparedness, response: available at http://www.who.int/csr/don/archive/country/en/ and Disease outbreaks: available at: http://www.who.int/topics/disease_outbreaks/en/. Accessed 15 October 2017. Probabilistic population projection for 2020 for all African countries using the exponential formula (https://en.wikipedia.org/wiki/List_of_African_countries_by_population) and the data from UN (DESA), 2017 (http://esa.un.org/unpd/wpp/. Accessed 1 November 2017).

**Figure 1:**
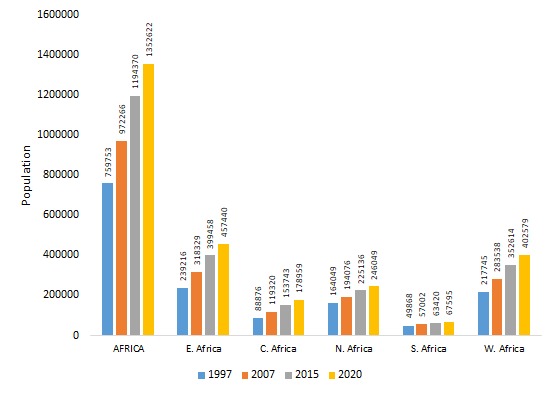
The Africa and sub-regional population trends in thousands (‘000), 1997-2020. Source: UN (DESA), 2017.

Human populations in African countries have rapidly increased in the last few decades (**Figure 1**). Population growth has occurred together with a substantial modification of human and pathogen behaviours [2,4]. For example, Hoosegood has identified that societal behaviours eg, union formation and cohabitation, re-marriage and partnering, union instability-widowhood, divorce and separation, fertility and fecundity, and fertility-related decisions significantly impact on and are impacted by HIV and AIDS in sub-Saharan regions particularly, in Southern Africa [5]. Second, urban growth has disrupted wildlife and pathogen ecology [1,2]. As human-pathogen contacts increase, so does the probability for more outbreaks. Pathogens affected include cowpox, Lyme disease, Nipah, Hendra and Ebola viruses as well as the group named ESKAPE –which includes Enterococci, *S. aureus*, *K*. *pneumoniae*, *A. baumannii*, *P. aeruginosa* and Enterobacteria – which explains between 45 and 77% of all African human mortalities [1,3]. As human-pathogen contacts increase, so does the probability of more outbreaks. To prevent epidemics, it is needed to markedly improve public infrastructures, sanitation, and the health systems. For instance, the public and animal health surveillance systems must transform, so interventions occur within acceptable response times [1,3,4].

Third, in the 20^th^ century, the average temperature has increased approximately 0.7°C in the African continent. Climate change has predisposed Africa to highly vulnerable situations, particularly around internationally shared water resources. Consequently, new challenges have emerged, including: border-related conflicts, food security risk due to declines agricultural production, vector- and water-borne diseases, (especially in areas with inadequate health infrastructure), flooding and exacerbation of desertification by changes in rainfall and intensified land use [2]. Predictions related to water resources include: (a) decreased rainfalls in portions of the Sahel, (b) increased rainfalls in east central Africa, (c) increased temperature ranging from 0.2°C to >0.5°C per decade, especially in the semi-arid margins of the Sahara and central southern Africa [6]. Some studies have also suggested that major climate change will influence water resource use, natural resources management and biodiversity, human health, food security, resettlement and infrastructure re-allocation, and desertification [2,6]. Variance in climatic conditions will impact significantly on disease ecology and epidemiology with upsurge in human-animal disease conditions due decreased salinity of the soil which can increase the number of toxic bacteria and breeding sites for mosquitoes and rodents.

These challenges can have consequences on international trade and commerce. Since the liberalization of trade policies between countries over the past two decades, national economies have grown in leaps and bounds. While such policies have fast-tracked growth forecast for African countries, they also have augmented the risks of emergent and trans-boundary animal and human diseases especially associated with long flights, such zoonotic tuberculosis, influenza viruses, HIV/AIDS and cholera. Because trans-border movements of livestock and/or some commercial practices may bring together disease vectors and humans, human and animal health should be addressed together [1-3].

Fourthly, the rapid expansion in human populations (and consequently, the need to meet the food security needs) has warranted the intensification of animal and crop agriculture. These changes have converted previously fallow lands and forest into arable, agricultural and/or grazing lands. Associated with these changes are increased (a) rodents populations, (b) dispersal and redistributions of wild ruminants populations and their ectoparasites, (c) wildlife-livestock-human interactions, and (d) occurrence of diseases like Rift Valley Fever. Bodies of evidence have suggested that the rate of future zoonotic diseases will be closely linked to the evolution of the agriculture-environment nexus [1,2]. It is suggested that, as long as Africa (or any other continent) does not address complex interactions –such as those that involve agriculture, the environment, economics, sociology, as well as zoonotic pathogens, disease outbreaks may follow human-driven disruptions, as those observed after major changes in land use, eg, those related with the construction of dams, mines, and intensive agriculture.

The fact that pathogens have evolved and keep evolving should be emphasized. Microbes such as *Mycobacterium tuberculosis, Enterococcus faecium, Enterobacter cloacae, Klebsiella pneumoniae, S. aureus, Acinetobacter baumanii* and *Pseudomonas aeruginosa* have developed multiple resistance mechanisms due to excessive and long-term use of antimicrobials, genetic transfers of resistance genes and selective pressures [2,3]. Endemic antimicrobial-resistant pathogens come with heavy clinical and economic burdens, especially in the developing countries. Recent review had indicated that endemic infections associated with antimicrobial resistance requires a particular attention because such diseases are linked with approximately 44 to 77% of all annual human deaths in Africa [3]. It has been estimated that, by 2050, more lives will be lost due to antimicrobial resistance (AMR) than cancer [3]. One major component of antimicrobial resistance is the overuse of antimicrobials in the production of livestock, which are then passed to humans [3]. Because vaccines reduce the incidence of infectious diseases (and, therefore, antibiotic use), immunisations might reduce AMR [1,3,7].

**Figure:**
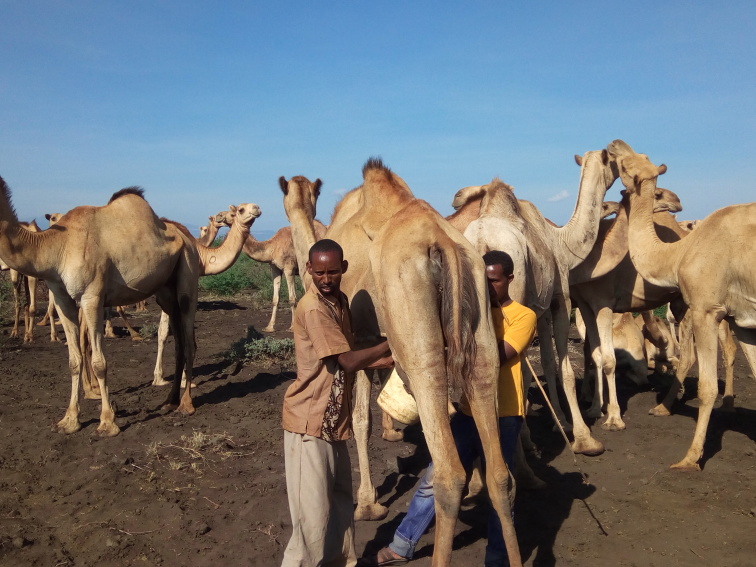
Photo: By Folorunso Fasina (Isiolo, Kenya). Intense human-animal interactions, and consumption of non-certified pathogen-free animal products facilitate the spread of zoonoses

Prevention and mitigations: given the numerous and serious issues here identified, African governments need to prioritize efforts aimed at rapid detection and prompt response to emerging or re-emerging pathogens. For example, the Critical Response Time (CRT or time available to implement effective epidemic control measures) should be considered in decision-making [1,5]. That is so because for any intervention to be very effective (≈ 100%), it must be deployed under a realist timeframe; if it requires a longer period of time, it will necessarily be (i) less effective (if not ineffective), and (ii) more expensive [5]. CRT may or may not include geo-referenced data. When it lacks geographical data, it becomes much shorter, making useless almost any intervention. Thus, the real significance of CRT is that it should be expanded (giving decision-makers more time to complete interventions) − which can only be achieved when high-resolution geo-referenced epidemiologic data are analyzed in time and space. Thus, to achieve improved epidemic control measures, geographically explicit data should be collected from epidemics, analysed and lessons learnt made available for future interventions. Only such (local or regional) data can support scientifically valid decision-making. Yet, even recent epidemics have been addressed with ad hoc policies, such as the classic ‘3-km radius control rings' – which assume all epidemics (ie, all pathogens, all host species, and all local geographies) are identical [1-3]. Failure to consider local bio-geo-epidemiological information has led to widespread dissemination of major epidemics, such as Ebola in Guinea, Liberia, and Sierra Leone [8].

Infectious diseases and zoonoses are extremely expensive to nations both clinically and economically, for example a recent valuation had estimated such costs to include: SARS in Asia and Canada (US$ 30-50 billion), HPAI H5N1 globally (US$ 30 billion), worldwide influenza H1N1 (US$ 45-55 billion), Ebola in West Africa (US$ 10 billion) and Zika in Latin America and the Caribbean (US$ 7-18 billion) [9], promptly delivered pre-emptive actions and control measures, as well as targeted interventions can significantly reduce burdens associated with these diseases.

The creation of interdisciplinary educational programs aimed at local and regional decision-makers involved in disease diagnosis, dissemination, and control, is recommended. Such programs could develop and integrate: (i) local data on antimicrobial resistance, (ii) high-resolution, local geo-referenced data, and (iii) site-specific control measures that can be implemented within biologically valid critical response times.
